# Gamified Physical Rehabilitation for Older Adults With Musculoskeletal Issues: Pilot Noninferiority Randomized Clinical Trial

**DOI:** 10.2196/39543

**Published:** 2023-03-06

**Authors:** Mirana Randriambelonoro, Caroline Perrin Franck, François Herrmann, Gorki Antonio Carmona, Antoine Geissbuhler, Christophe Graf, Emilia Frangos

**Affiliations:** 1 HI5Lab, Department of Radiology and Medical Informatics Faculty of Medicine University of Geneva Geneva Switzerland; 2 Division of Rehabilitation and Geriatrics Geneva University Hospitals University of Geneva Geneva Switzerland

**Keywords:** rehabilitation, gamification, emerging technologies, experimental, randomized controlled trial, mobility, device, musculoskeletal, older patients, elderly, aging, functionality, physical therapy, computer-aided, intervention, serious games

## Abstract

**Background:**

Resource-rich countries are facing the challenge of aging societies, a high risk of dependence, and a high cost of care. Researchers attempted to address these issues by using cost-efficient, innovative technology to promote healthy aging and regain functionality. After an injury, efficient rehabilitation is crucial to promote returning home and prevent institutionalization. However, there is often a lack of motivation to carry out physical therapies. Consequently, there is a growing interest in testing new approaches like gamified physical rehabilitation to achieve functional targets and prevent rehospitalization.

**Objective:**

The purpose of this study is to assess the effectiveness of a personal mobility device compared with standard care in the rehabilitation treatment of patients with musculoskeletal issues.

**Methods:**

A total of 57 patients aged 67-95 years were randomly assigned to the intervention group (n=35) using the gamified rehabilitation equipment 3 times a week or to the control group (n=22) receiving usual standard care. Due to dropout, only 41 patients were included in the postintervention analysis. Outcome measures included the short physical performance battery (SPPB), isometric hand grip strength (IHGS), functional independence measure (FIM), and the number of steps.

**Results:**

A noninferiority related to the primary outcome (SPPB) was identified during the hospital stay, and no significant differences were found between the control and intervention groups for any of the secondary outcomes (IHGS, FIM, or steps), which demonstrates the potential of the serious game-based intervention to be as effective as the standard physical rehabilitation at the hospital. The analysis by mixed-effects regression on SPPB showed a group×time interaction (SPPB_I_t1=–0.77, 95% CI –2.03 to 0.50, *P*=.23; SPPB_I_t2=0.21, 95% CI –1.07 to 0.48, *P*=.75). Although not significant, a positive IHGS improvement of more than 2 kg (Right: 2.52 kg, 95% CI –0.72 to 5.37, *P*=.13; Left: 2.43 kg, 95% CI –0.18 to 4.23, *P*=.07) for the patient from the intervention group was observed.

**Conclusions:**

Serious game-based rehabilitation could potentially be an effective alternative for older patients to regain their functional capacities.

**Trial Registration:**

ClinicalTrials.gov NCT03847454; https://clinicaltrials.gov/ct2/show/NCT03847454

## Introduction

A globally growing geriatric population emphasizes the importance of providing a healthy aging environment [1]. Rather than the absence of disease, “healthy aging” is defined as a process that enables older people to continue to perform activities of daily living and maintain social contact [2-4]. However, as people age, the prevalence of chronic conditions increases. Due to their polymorbidity, older adults are hospitalized longer and more frequently than younger ones, increasing their risk of functional decline [5]. Therefore, after an acute health problem, a rehabilitation phase is often required to regain functionality before returning home.

Musculoskeletal disorders are one of the main reasons for geriatric hospitalization in Switzerland [6], affecting joints, bones, and muscles. During their hospital stay, patients with musculoskeletal issues follow rehabilitation therapy to regain physical function and the capacity to perform daily tasks such as standing, walking, climbing stairs, or bathing independently. Functionality at discharge is inversely proportional to the risk of rehospitalization [7]. After functional recovery, the hospital-to-home transition is increasingly recognized as a critical period, notably to prevent further functional decline and rehospitalizations [8]. Regular physical activity remains the central point to influence these 2 outcomes, but it needs motivation to be maintained over time [9-11].

Researchers have extensively studied the use of computer-aided physical rehabilitation to promote physical activity. Taylor et al [12] performed a meta-analysis to systematically evaluate whether active video games could improve measures of physical performance in older adults and found positive results related to the improvement of mobility and balance. Idris et al [13] developed specific game scenarios, evaluated them with a panel of patients with musculoskeletal issues, and showed the usefulness of the guidelines and associated games. Serious games coupled with monitoring devices such as Kinect [14] have shown the potential to positively impact patients’ motivation to perform rehabilitation exercises [13,15]. The use of a gamified rehabilitation system in addition to or instead of standard physical rehabilitation will have several potential advantages for the health system, the health professionals, and the patients, such as lower hospital costs, shorter hospital stays, and better access to care.

However, whether such devices would be as effective as standard care rehabilitation in the hospital in engaging older adults to remain active after discharge is still understudied.

The objective of the trial was to compare the effectiveness of a gamified rehabilitation device with the standard of care to help older adult patients regain their functional capacities and maintain them 3 weeks after discharge. We already demonstrated that such an approach improved motivation for therapies in a qualitative paper based on the same study, where the focus was more on acceptance, motivation, and engagement [16]. Our hypothesis is that patients in the intervention group will regain independence as much as those in the control group in terms of strength, speed, and balance and that their abilities will be maintained over time.

## Methods

### Participants

The study took place at 2 different sites in Switzerland: Loëx Hospital, a 104-bed geriatric post-acute rehabilitation hospital, and Joli-Mont, a 60-bed geriatric rehabilitation clinic. Both are part of the Geneva University Hospitals, where the participants’ recruitment took place.

The eligibility criteria were stipulated as follows: patients (aged 65 years and older) hospitalized in one of the 2 study sites with musculoskeletal issues (pelvic or lower limb fractures, hip prostheses, falls, and low back pain), able to stand upright, and capable of understanding the instructions. Being able to interact with the equipment without any sensory, physical, or mental limitations was necessary. Patients considered too weak to interact with the device or planning to go to a nursing home were excluded. Due to a limitation associated with the device’s size, patients with obesity were not eligible.

### Study Design

The study is a 2-arm multicenter noninferiority randomized clinical trial examining the effectiveness of gamified rehabilitation equipment to improve older adults’ functional capacities.

The hospital’s electronic medical records of all newly hospitalized patients were accessed (from February to June 2019) to identify potential participants. All patients fulfilling the eligibility criteria were approached by the researchers. If the patient agreed to participate, researchers asked the patient to sign an informed consent form. Participants were then allocated randomly to one of the 2 arms of the trial. The randomization was based on a single allocation ratio, with no block and no stratification. Due to the type of intervention, the allocation was not masked to the participants in the intervention and control groups or to the researchers who recruited the participants.

### Materials

**Figure 1:**
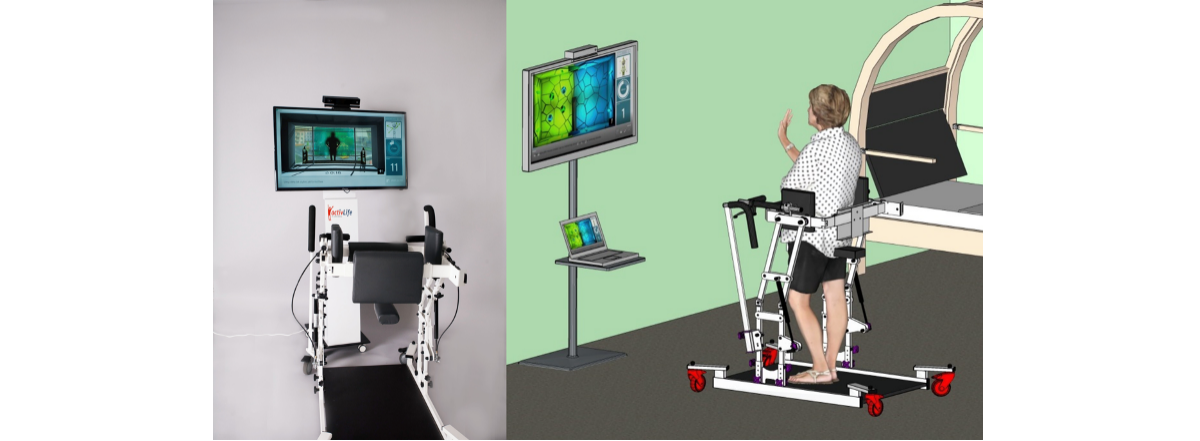
ActivLife.

**Figure 2:**
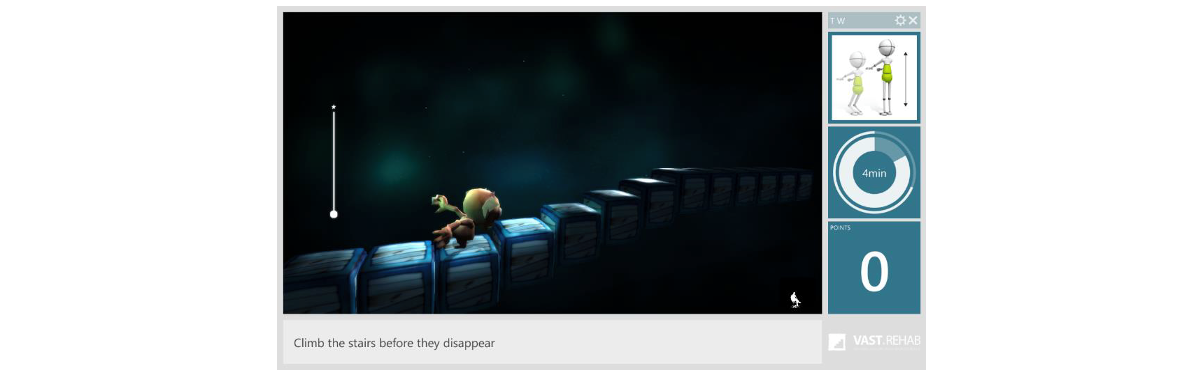
Patient’s interface for “Stairs” game.

**Figure 3:**
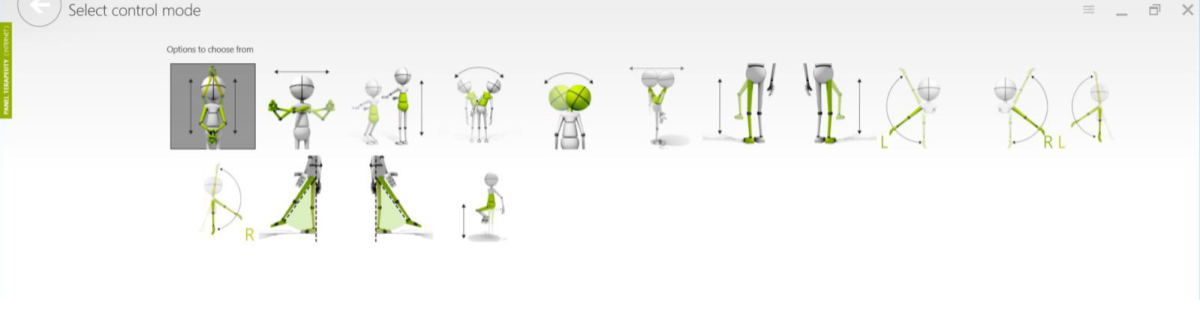
Physiotherapist’s interface—control mode selection.

ActivLife (Figure 1) is a multifunctional rehabilitation equipment system with different functionalities such as physical activation, rehabilitation, mobility, bed assistance (eg, transfer), and mental stimulation. The equipment is coupled with a serious game platform called Vast.Rehab, which allows the patients to complete their exercises (lower limbs, upper limbs, or both) while playing games. In addition to the game components, ActivLife is composed of an efficient trunk stabilization that reassures the patients while engaging in different movements such as “cleaning the window,” “guiding an ambulance,” or “flying a dragon” [17]. Figure 2 illustrates an example of a game (the “Stairs“ game). The games and instructions are displayed on a screen in front of the patient, who is secured in the ActivLife mechanical platform. The screen has a Kinect sensor that allows the software to determine if the patient is doing the exercise correctly. The software allows the physiotherapist to program and schedule a specific treatment (a series of games) for each patient. Based on the patient’s ability and progress, the physiotherapist can easily adjust the type of movement (Figure 3) to control the game as well as the level of difficulty (by defining the required range of motion for each movement).

The game called “Stairs” is about the creature jumping on the stairs one at a time. To make the creature jump, the patient needs to do a sit-up. The range of movement can be adjusted to the capabilities of the patient.

**Figure 4:**
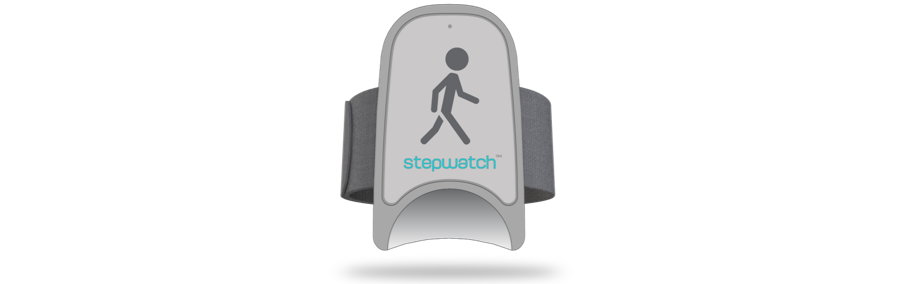
Stepwatch.

Stepwatch (Figure 4) [18] is a small (75 mm × 48 mm × 14 mm) and light (41 g) tri-axial accelerometer that can measure the activity of the patient in terms of the number of steps, activity (low, medium, and high), cadence, and velocity. It has a sampling frequency of 200 Hz, and data can be available in 1-second epochs. The wearable does not display any information and can be worn on the ankle using a Velcro strap. The Stepwatch can capture small changes in step rate (99% accuracy [19,20]), thus it can be used to assess changes in physical activity in individuals who walk slowly or use a walking aid such as a rollator. Furthermore, it allows local data collection, which ensures patient privacy.

### Procedure

**Figure 5:**
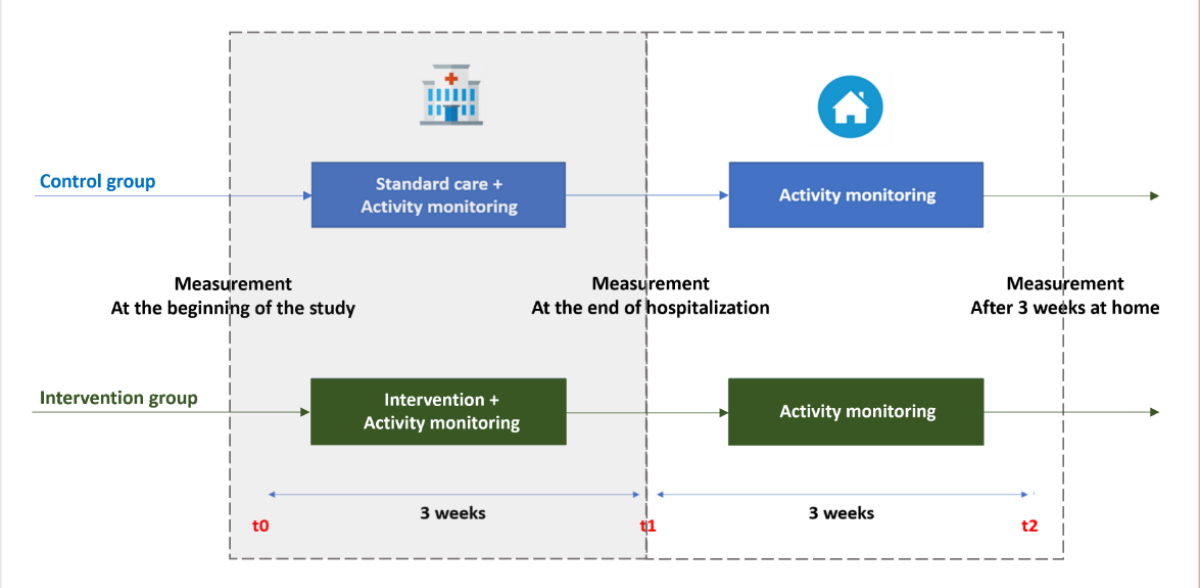
Research procedure.

For 3 weeks, both intervention and control groups participated in 30-minute training sessions 5 times a week. The intervention group used ActivLife 3 times a week during these sessions, while the control group had all their sessions consist of standard physical therapy sessions. During their hospital stay, the rehabilitation was performed under the supervision of 2 physiotherapists (one at each site). The games played by the patients in the intervention group were selected and defined by the physiotherapist based on the patient’s treatment needs and abilities. If the patient needed to do an upper limb exercise (eg, moving the right hand up and down, making a 45-degree angle), the physiotherapist was able to choose this movement to control the game. The patients could then play a series of games defined by the physiotherapist during their hospital stay. After discharge (week 3), the patients were assessed at home by the physiotherapist at week 6 (Figure 5). Depending on their state at discharge, some of the patients were recommended to continue physiotherapy at home. All participants in both groups wore the Stepwatch sensor during the 6 weeks.

### Primary and Secondary Outcomes

Used as a primary outcome, the short physical performance battery (SPPB) is an objective tool for assessing lower extremity functioning in older people [21]. This test is associated with the risk of falls, the risk of functional decline, and the risk of death [22-24]. The test consists of 3 parts: balance tests, gait speed tests, and chair stand tests. The SPPB test is based on a point system, with a maximal score of 12 points, meaning an ability to function independently.

As secondary outcomes, we measured:

1. Isometric hand grip strength (IHGS) is a simple and cost-effective method for evaluating overall muscle strength [25]. It is associated with cardiovascular mortality and is a main determinant of sarcopenia (a condition characterized by progressive and generalized loss of skeletal muscle mass and strength) [26,27]. The participant is asked to hold the dynamometer in the hand to be tested, with the arm at a right angle and the elbow next to the body. He or she is asked to tighten the dynamometer with maximum isometric effort, which is maintained for about 5 seconds. The score is expressed in kilograms.
2. Functional independence measure (FIM), as a basic indicator of the degree of functionality. This score is associated with the risk of rehospitalization [28]. It is composed of 18 different items scored from 1 (complete assistance required) to 7 (complete independence). The FIM is used to assess functionality in 6 areas, including self-care, continence, mobility, transfers, communication, and social cognition [29]. The FIM is based on a point system, with a maximum of 126 points, meaning an ability to function independently.
3. The number of daily steps, assessed by Stepwatch. Patient data were collected at 3 different times: at baseline (t0), after the intervention (t1—end of hospitalization), and 3 weeks after returning home (t2). The SPPB test and the IHGS test were conducted at times t0, t1, and t2. FIM was evaluated at times t0 and t1. Baseline data included age, gender, the Cumulative Illness Rating Score, and the Mini Mental State Evaluation [30,31].

### Statistics

The study is a noninferiority trial to test if the gamified rehabilitation concept is at least as effective as standard care with respect to the main outcome measured by SPPB. With a power calculation of 95%, a mean (SD) of 8.8 (1.2), and a noninferiority limit of 0.4, the total sample size needed was 38. Adjusting for a dropout rate of 20%, the sample size needed was increased to 46 patients in total, 23 in each group. To recruit this number of patients, a 6-month inclusion period was anticipated.

The characteristics of subjects are presented as mean (SD) for continuous variables. The normality of the distribution of continuous variables was verified with Shapiro-Wilks tests. We used a 2-sample *t* test and Fisher exact test to compare baseline data. Mixed-effects multiple linear regression models were used to assess the group and time effects and their interaction on the outcome while taking into account the repeated measure design and adjusting for the presence of a physiotherapist at home and other variables such as the number of sessions, age, and gender. The difference-value was considered significant when *P*<.05. Statistical analyses were performed with STATA (version 16.0; StataCorp).

### Ethics Approval and Trial Registration

The study has been approved by the Commission Cantonale d'Ethique de la Recherche (CCER) (number 2018-01516). The trial has been registered in the register ClinicalTrials.gov (NCT03847454).

## Results

### Patients Flow Diagram

**Figure 6:**
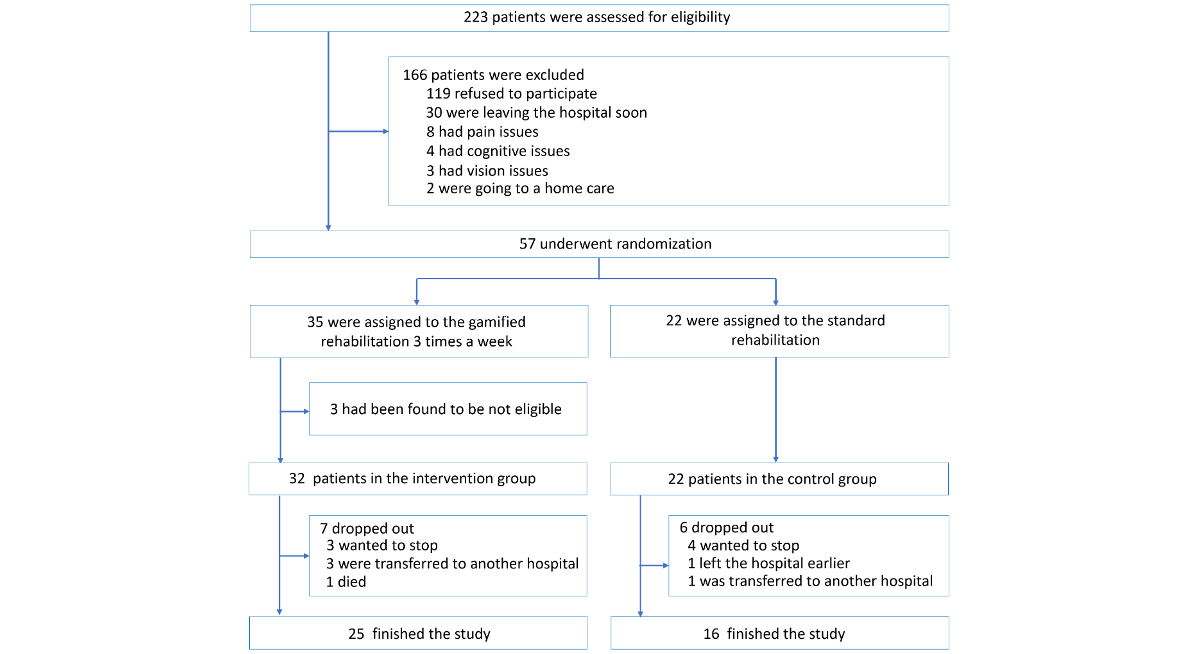
Patients flow diagram.

The patients flow diagram is described in Figure 6. A total of 223 patients were screened for eligibility. Of these, 166 were excluded from the study (119 refused to participate, 30 were leaving the hospital shortly, 8 had pain issues, 4 had cognitive issues, 3 had vision issues, and 2 were going to a care home). A total of 57 patients underwent randomization to be allocated to the intervention group (n=35) and the control group (n=22) and were included in the main analysis. During the follow-up phase, 10 patients dropped out from the intervention group and 6 patients from the control group.

### Baseline Data

The mean age of the total participants was 81.5 (SD 6.8) years, with 68.4% (39/57) female participants. The length of stay at the hospital was 23.0 (SD 11.6) days on average. The Cumulative Illness Rating Score scored 14.3 (SD 6.4) on average. The Mini-Mental State Evaluation showed a mean value of 23.4 (SD 5.1). The SPPB showed a mean of 6.36 (SD 2.8) at baseline. FIM at admission was 97.4 (SD 16.1) on average. The IHGS scored 20.7 (SD 9.3) kg on average on the right hand and 21.0 (SD 8.6) kg on average on the left hand. The mean number of steps was 1402 (SD 1162) steps. Participants’ data at baseline are described in Table 1. Comparisons of the groups at baseline showed no evidence of differences between the groups in any of the measures.

**Table 1:** Patients’ baseline data (For the FIM^a^, CIRS^b^, MMSE^c^, SPPB^d^, IHGS^e^, and steps, higher is better).

	Total (n=57)	Intervention group (n=35)	Control group (n=22)	*t* test (*df*)	*P* value
Age (years), mean (SD)	81.5 (6.8)	82.2 (7.0)	81.5 (6.8)	0.37 (55)	.71
Female gender, n (%)	39 (68.42)	24 (68.57)	15 (68.18)	0.48 (55)	.63
Length of stay (days), mean (SD)	23.0 (11.6)	22.5 (11.1)	23.8 (12.7)	–0.40 (55)	.68
FIM (score), mean (SD)	97.4 (16.1)	98.7 (15.2)	95.3 (17.6)	0.77 (55)	.44
CIRS (score), mean (SD)	14.3 (6.4)	14.4 (5.7)	14.1 (7.5)	0.17 (55)	.86
MMSE (score), mean (SD)	23.4 (5.1)	23.6 (6.1)	23.1 (3.7)	0.35 (55)	.73
SPPB (score), mean (SD)	6.36 (2.78)	6.23 (2.80)	6.58 (2.83)	–0.46 (55)	.65
IHGS right (score), mean (SD)	20.66 (9.3)	19.56 (8.67)	21.77 (9.93)	–0.88 (55)	.38
IHGS left (score), mean (SD)	21 (8.57)	19.71 (8.60)	22.29 (8.54)	–1.11 (55)	.27
Steps (score), mean (SD)	1402 (1162)	1359 (1047)	1468 (1319)	–0.36 (55)	.73
Physiotherapy at home, n (%)	30 (52.63)	17 (48.57)	13 (59.09)	0.28 (55)	.78

^a^FIM: functional independence measure.

^b^CIRS: Cumulative Illness Rating Score.

^c^MMSE: Mini-Mental State Evaluation.

^d^SPPB: short physical performance battery.

^e^IHGS: isometric hand grip strength.

### Outcomes

#### Overview

**Figure 7:**
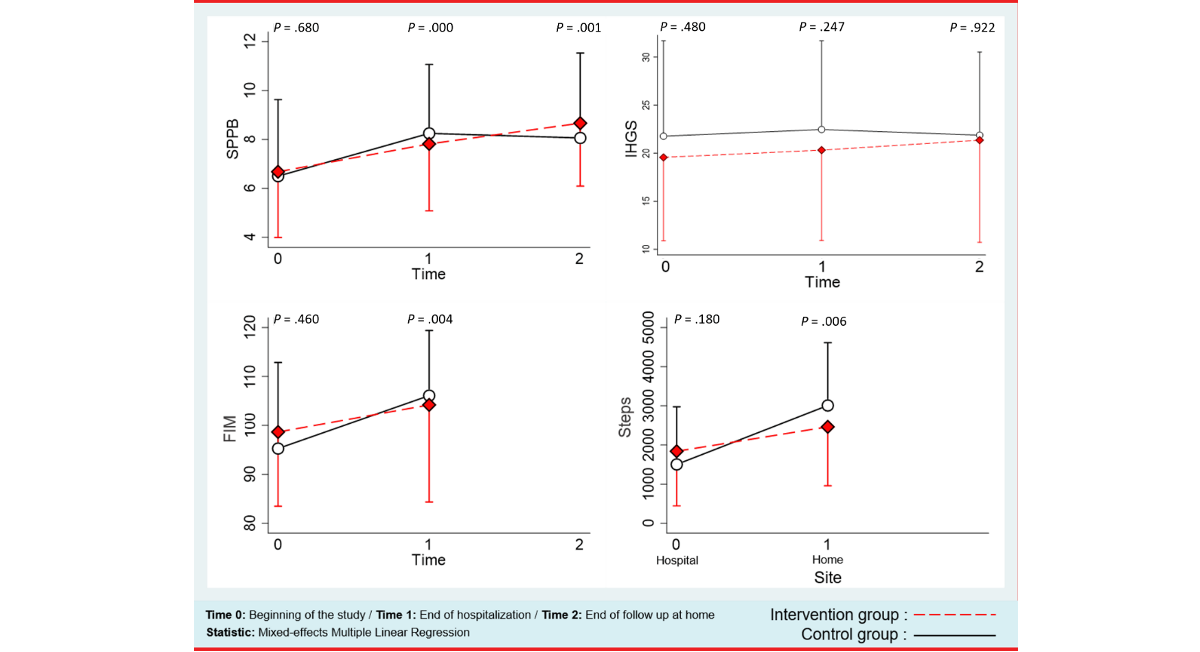
Outcomes: SPPB, IHGS—right hand, FIM, and steps. FIM: functional independence measure; IHGS: isometric hand grip strength; SPPB: short physical performance battery.

Figure 7 and Table 2 summarize the outcomes. The analysis by mixed-effects regression on the primary outcome (SPPB) showed a groupxtime interaction (SPPB_I_t1=–0.77, 95% CI –2.03 to 0.50, *P*=.23; SPPB_I_t2=0.21, 95% CI –1.07 to 0.48, *P*=.75) during hospitalization and at home. Due to our small sample size, the wide CIs made our results inconclusive for most of the defined outcomes. However, although not significant, the group×time interaction between t0 and t1 (SPPB_I_t1=–0.77, 95% CI –2.03 to 0.50, *P*=.23) was <0.4 (noninferiority margin). Additionally, no significant differences in any of the secondary outcomes (IHGS, FIM, or steps) were found between the control and the intervention groups.

**Table 2:** Results of mixed-effects regressions.

Outcome variable	SPPB^a^		IHGS^b^ (right)		IHGS (left)		FIM^c^		Number of steps	
	Coefficient (95% CI)	*P* value	Coefficient (95% CI)	*P* value	Coefficient (95% CI)	*P* value	Coefficient (95% CI)	*P* value	Coefficient (95% CI)	*P* value
Age	–0.10 (–0.21 to 0.01)	.07	–0.30 (–0.56 to –0.04)	.02	–0.23 (–0.53 to 0.06)	.12	–0.62 (–.15 to –0.10)	.02	–52 (–88 to –16)	.004
Gender(male)	1.37 (–0.19 to 2.93)	.09	12.22 (8.62 to 15.83)	.30	12.78 (8.48 to 17.09)	.60	0.86 (–6.64 to 8.36)	.82	513 (–27 to 1053)	.06
Hospital	0.54 (–1.22 to 2.30)	.55	6.44 (–0.10 to 12.97)	.05	5.80 (–0.17 to 11.78)	.06	N/A^d^	N/A	–692 (–1205 to –178)	.008
Intervention group	–0.65 (–3.12 to 1.83)	.61	–2.62 (–8.31 to 3.08)	.37	–0.23 (–8.63 to 2.16)	.24	4.47 (–4.07 to 13.0)	.31	23 (–664 to 711)	.95
Time 1	1.79 (0.81 to 2.78)	<.001	1.22 (–0.84 to 3.28)	.25	1.24 (–0.31 to 2.80)	.12	10.82 (3.40 to 18.23)	.004	N/A	N/A
Time 2	1.60 (0.62 to 2.59)	.001	–0.12 (–2.62 to 2.37)	.92	0.33 (–1.40 to 2.05)	.71	N/A	N/A	740 (5 to 1474)	.048
Interaction time 1	–0.77 (–2.03 to 0.50)	.23	–0.21 (–2.81 to 2.39)	.88	–0.40 (–2.39 to 1.58)	.69	–5.26 (–14.85 to 4.32)	.28	N/A	N/A
Interaction time 2	0.21 (–1.07 to 1.48)	.75	2.33 (–0.72 to 5.37)	.14	2.03 (–0.18 to 4.23)	.07	N/A	N/A	505 (–448, 1458)	.30
Physio at home	2.43 (0.55 to 4.31)	.01	2.50 (–4.27 to 9.27)	.47	0.94 (–5.51 to 7.40)	.78	N/A	N/A	N/A	N/A
Number of sessions	0.17 (–0.12 to 0.46)	.25	N/A	N/A	N/A	N/A	N/A	N/A	N/A	N/A

^a^SPPB: short physical performance battery.

^b^IHGS: isometric hand grip strength.

^c^FIM: functional independence measure.

^d^N/A: not available.

#### Primary Outcome

Regarding SPPB, there was a main effect of time (SPPB_t1=1.79, 95% CI 0.81-2.78, *P<.*001; SPPB_t2=1.60, 95% CI 0.62-2.59, *P=.*001) reflecting the overall improvement in SPPB score across the 3 measurement points. The main effect of having physiotherapy at home (SPPB=2.43, 95% CI 0.55-4.31, *P*=.011) indicated that remaining active at home has a positive effect on the SPPB score. Although not significant (SPPB_nb_of_sessions=0.17, 95% CI –0.12 to 0.46, *P=.*248), the effect of the number of sessions on the machine tended to be positive. A main effect of gender was also observed (SPPB=1.37, 95% CI –0.19 to 2.93, *P*=.09) indicating that male patients are more active.

#### Secondary Outcomes

We observed an improvement of more than 2 kg (Right: 2.33 kg, *P*=.13; Left: 2.03 kg, *P*=.07) of IHGS in the intervention group. For the FIM, there was also a main effect of time (FIM_t1=10.8, *P*=.004), reflecting the overall improvement in the FIM score between the 2 measurement points. The mean number of steps (Steps_I=1839; Steps_C=1504) showed that participants in the intervention were somewhat more active at the hospital compared to the control group. However, at home, we failed to observe the same results (Steps_I=2463; Steps_C=3008), while there was a main effect of the site (Steps_hospital=–692, *P*=.008; Steps_home=740, *P*=.048) reflecting an improvement in the number of steps while returning home.

## Discussion

### Principal Findings

We evaluated the effectiveness of ActivLife, a gamified rehabilitation equipment, for improving functional capacities among older adults with musculoskeletal issues and maintaining them over time. A noninferiority related to the primary outcome (SPPB) was identified during the hospital stay (although it was not significant), and no significant differences were found between the control and intervention groups for any of the secondary outcomes (IHGS, FIM, or steps). These results show the potential of the serious game-based intervention to be as effective as the standard rehabilitation at the hospital.

### Comparison to Prior Work

The potential of serious games to improve overall health and specific disease management in older adults has been explored intensively. Parkinson disease [32-34] and stroke rehabilitation [35-37] have been topics of interest for gamified intervention developers. However, a literature review on Kinect-based stroke rehabilitation systems [38] illustrates that previous studies were driven more toward the feasibility and technical effectiveness of such systems than their clinical effectiveness. A similar observation has been found in the use of gamification for cognitive assessment and cognitive training [39].

Additionally, although not statistically significant, it is worth noting that, after 6 weeks, the handgrip strength test improved by 2 kg in the intervention group compared to 0.3 kg in the control group. This effect was likely due to the fact that ActivLife encourages safe upper limb exercises. Knowing that an improvement of 2 kg is considered a minimally significant change in the handgrip test [40], this result demonstrates the potential of gamified rehabilitation to maximize the improvement of older adult patients’ muscle strength.

Furthermore, if proven to be as effective as standard care, gamified rehabilitation could potentially induce cost-effectiveness by reducing the time spent by the physiotherapist with the person during a therapy session. Such tools could enable the physiotherapist to manage multiple patients simultaneously, requiring only passive surveillance instead of actively monitoring each one of them. A cost-effectiveness analysis conducted by Rongbo [41] on the use of an intelligent bed system coupled with ActivLife at the hospital showed that relying on the equipment would reduce the time spent by the physiotherapist on one patient from 6 to 2 hours. This would reduce considerably the burden of limited health professionals associated with the increase of musculoskeletal disorders and the prevalence of the aging population [42]. However, as patients value patient-therapist interaction more than the amount or content of therapy during inpatient rehabilitation [43], further investigation is needed to understand the trade-off between those 2 components.

### Limitations, Strengths, and Future Directions

Our study presents several limitations. First, the sample size of the study was small, making it difficult to detect moderate effects (eg, differences between groups), especially as we observed several variabilities in the steps’ data. Second, due to the subsequent dropouts, some data were missing. Analyses of postintervention results were then adjusted for the remaining participants (n=41). Third, the limitations associated with the length of stay of the patients made it difficult to ensure that the intervention group had enough sessions on the machine. However, this experiment also has multiple strengths. First, although based on a small sample size, our study has the benefit of investigating the clinical validity of serious game-based rehabilitation in a real-world setting. Second, the 3-week follow-up at home allowed us to get an overview of patients’ improvement after leaving the hospital. To further validate this study, the inclusion of a larger sample size for a longer period is necessary. Another interesting direction could be about understanding and evaluating the potential of using gamified rehabilitation equipment as a hospital-to-home transition tool where the patient will continue to have access to the system (eg, via social institutions) even after discharge.

### Conclusions

Our pilot study demonstrated the potential of the ActivLife device, a gamified rehabilitation equipment, to be as effective as standard care (noninferiority) in the treatment of older adults with musculoskeletal issues.

## Abbreviations

FIM: functional independence measure
IHGS: isometric hand grip strength
SPPB: short physical performance battery

## Data Availability

The data sets generated or analyzed during this study are available from the corresponding author upon reasonable request.
